# Numerical and Series Solutions for Stagnation-Point Flow of Nanofluid over an Exponentially Stretching Sheet

**DOI:** 10.1371/journal.pone.0061859

**Published:** 2013-05-09

**Authors:** Meraj Mustafa, Muhammad A. Farooq, Tasawar Hayat, Ahmed Alsaedi

**Affiliations:** 1 Research Centre for Modeling and Simulation, National University of Sciences and Technology, Islamabad, Pakistan; 2 Centre for Advanced Mathematics and Physics, National University of Sciences and Technology, Islamabad, Pakistan; 3 Department of Mathematics, Quaid-I-Azam University, Islamabad, Pakistan; 4 Department of Mathematics, Faculty of Science, King Abdulaziz University, Jeddah, Saudi Arabia; University of California, Berkeley, United States of America

## Abstract

This investigation is concerned with the stagnation-point flow of nanofluid past an exponentially stretching sheet. The presence of Brownian motion and thermophoretic effects yields a coupled nonlinear boundary-value problem (BVP). Similarity transformations are invoked to reduce the partial differential equations into ordinary ones. Local similarity solutions are obtained by homotopy analysis method (HAM), which enables us to investigate the effects of parameters at a fixed location above the sheet. The numerical solutions are also derived using the built-in solver bvp4c of the software MATLAB. The results indicate that temperature and the thermal boundary layer thickness appreciably increase when the Brownian motion and thermophoresis effects are strengthened. Moreover the nanoparticles volume fraction is found to increase when the thermophoretic effect intensifies.

## Introduction

There has been great interest of researchers in the flow and heat transfer characteristics due to the impulsive motion of stretching sheet. A variety of technical processes involve the production of sheeting material which includes both metal and polymer sheets. The rate of heat transfer at the sheet is largely dependent on the quality of final product.

Flow past a flat plate with a uniform free stream was reported by Blasius [1]. In contrast to the Blasius problem, the boundary layer flow over a continuously moving plate in a quiescent ambient fluid was explored by Sakiadis [2]. Crane [3] extended this concept for a sheet which is stretched with the velocity linearly proportional to the distance from the origin. Since this pioneering work of Crane [3], the literature concerning the boundary layer flows past a stretching sheet has been in continuous growing. In fact Crane's problem has been considered for several other features such as viscoelasticity, heat and mass transfer, porosity, magnetic field etc. (see Rajagopal et al. [4], Mahapatra and Gupta [5], Cortell [6], Bachok et al. [7], Abbasbandy and Ghehsareh [8], Fang et al. [9], Hayat et al. [10], Mustafa et al. [11], [12] etc.). On the other hand, a literature survey witnesses that the flow analysis over an exponentially stretching sheet has been scarcely presented. Combined heat and mass transfer in the boundary layer flow over an exponentially stretching sheet has been reported by Magyari and Keller [13]. Suction and heat transfer characteristics in the exponentially stretched flow has been studied by Elbashbeshy [14]. Viscoelastic effects in the flow over an exponentially stretching sheet have been described by Khan and Sanjayanand [15]. Analytic solutions for flow and heat transfer over an exponentially stretching sheet have been provided by Sajid and Hayat [16]. Nadeem et al. [17] examined the flow and heat transfer of viscoelastic (second grade) fluid over an exponentially sheet in the presence of thermal radiation.

Nanofluid is a liquid suspended with nanometer-sized particles (diameter less than 50 nm) called nanoparticles. These nanoparticles are typically made of metals, oxides and carbides or carbon nanotubes. In the past, the concept of nanofluids has been used as a route to enhance the performance of heat transfer rate in liquids. Detailed review studies on nanofluids have been conducted by Daungthongsuk and Wongwises [18], Wang and Mujumdar [19], [20] and Kakaç and Pramuanjaroenkij [21]. Natural convective boundary layer flow of nanofluid past a vertical flat plate has been studied by Kuznetsov and Nield [22]. The Cheng-Mincowcz problem for flow of nanofluid embedded in a porous medium has been considered by Nield and Kuznetsov [23]. Bachok et al. [24] examined the flow of nanofluid over a continuously moving surface with a parallel free stream. Flow of nanofluid over a linearly stretching sheet has been studied by Khan and Pop [25]. Finite element analysis for flow of nanofluid over a nonlinearly stretching sheet is presented by Rana and Bhargava [26]. Falkner-skan problem for flow of nanofluid with with prescribed surface heat flux is investigated by Yacob et al. [27]. Makinde and Aziz [28] discussed the effect of convective boundary conditions on the flow of nanofluid over a stretching sheet. Analytic solutions for stagnation-point flow of nanofluid over a linearly stretching sheet are obtained by Mustafa et al. [29].

It is noticed that flow of nanofluid over an exponentially stretching sheet is never reported in the literature. Thus current work presents a theoretical study on the stagnation-point flow of nanofluid over an exponentially stretching sheet. The series expressions of velocity, temperature and nanoparticles concentration are developed by homotopy analysis method (HAM) developed by Liao [30]. This method is successfully applied to derive analytic solutions of variety of nonlinear problems [30]–[35]. The numerical solutions are obtained by the software MATLAB. Graphical results for various values of the parameters are presented to gain thorough insight towards the physics of the problem. The numerical values of reduced Nusselt number and reduced Sherwood number for different values of the parameters are also tabulated.

## Mathematical Formulation

We investigate the laminar boundary layer flow of a nanofluid in the region of stagnation-point towards an exponentially stretching sheet situated at 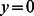. The 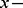 and 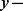 axis are taken along and perpendicular to the sheet and the flow is confined to 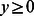. The effects of Brownian motion and thermophoresis are also accounted. 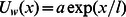 denotes the velocity of the sheet while the velocity of the external flow is 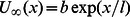. Let 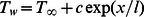 and 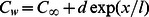 be the temperature and nanoparticles concentration at the sheet where 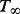 and 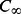 denote the ambient temperature and concentration respectively. The boundary layer equations governing the conservation of mass, momentum, energy and nanoparticles volume fraction are (see Kuznetsov and Nield [22], Nield and Kuznetsov [23], Bachok et al. [24], Khan and Pop [25], Rana and Bhargava [26], Yacob et al. [27], Makinde and Aziz [28] and Mustafa et al. [29])
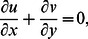
(1)

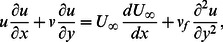
(2)

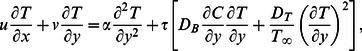
(4)

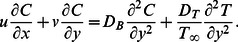
(5)With the boundary conditions
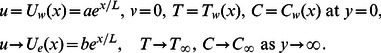
(6)Where 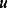 and 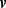 are the velocity components along 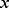
- and 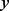
- directions respectively, 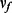 is the kinematic viscosity, 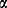 is the thermal diffusivity, 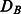 is the Brownian motion coefficient, 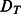 is the thermophoretic diffusion coefficient and 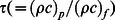 is the ratio of effective heat capacity of the nanoparticle material to heat capacity of the fluid.

We introduce
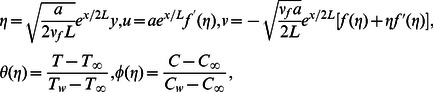
(7)


Inserting Eq. (7) into Eqs. (2)–(5) yield the following ordinary differential equations
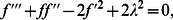
(8)

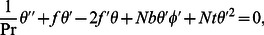
(9)

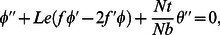
(10)

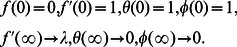
(11)

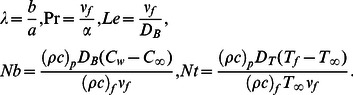
(12)


Here 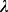 is the velocity ratio, 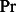 is the Prandtl number, 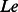 is the Lewis number, 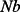 is the Brownian motion parameter and 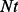 is the thermophoresis parameter. It is clear that 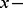
coordinate can not be eliminated from Eqs. (9) and (10) because 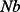 and 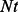 are functions of 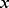. Thus we look for the availability of local similarity solutions which permits us to investigate the behaviors of these parameters at a fixed location above the sheet. 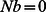 corresponds to the case when there is no thermal transport generated by the nanoparticles concentration gradients.

The skin friction coefficient 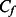, the local Nusselt number 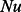 and the local Sherwood number 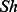 are given by
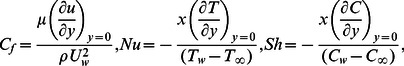
(13)Using (7) in (13) one obtains
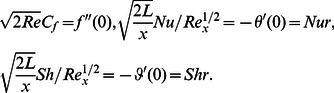
(14)where 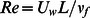 is the Reynolds number and 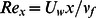 denotes the local Reynolds number.

## Methods of Solution

### 3.1 Homotopy analytic solution

Rule of solution expression and the involved boundary conditions direct us to choose the following initial guesses 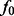, 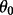 and 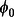 of 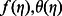 and 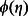


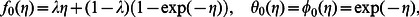
(15)The auxiliary linear operators are chosen as
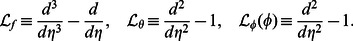
(16)


If 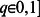 denotes the embedding parameter and 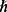 is the non-zero auxiliary parameter then the generalized homotopic equations are constructed as follows:
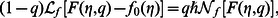
(17)

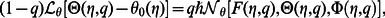
(18)

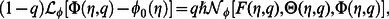
(19)

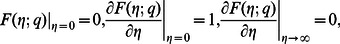
(20)

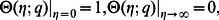
(21)

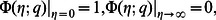
(22)in which the non-linear operators 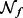, 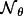 and 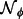 are
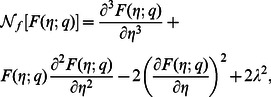
(23)

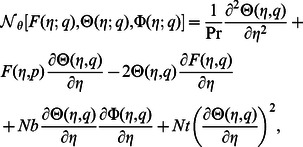
(24)

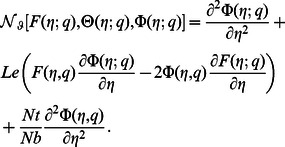
(25)


Using Maclaurin's series about 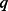


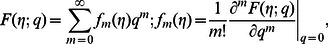
(26)

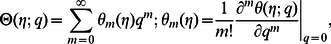
(27)

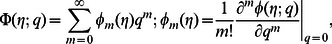
(28)the final solutions are retrieved at 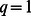. The functions 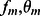 and 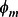 can be obtained from the deformation of Eqs. (17)–(22). Explicitly the deformation problems corresponding to Eqs. (17)–(22) are
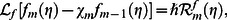
(29)

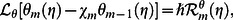
(30)

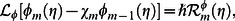
(31)

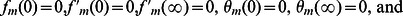


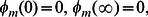
(32)

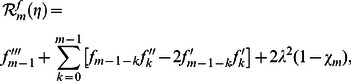
(33)

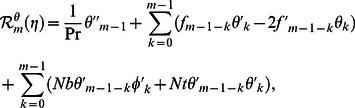
(34)

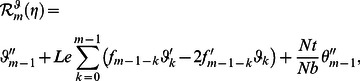
(35)

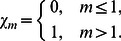
(36)


Eqs. (29)–(36) can be easily solved by using the symbolic computation software **Mathematica** for 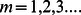


#### 3.1.1 Error analysis and convergence of the homotopy solutions

**Figure 1:**
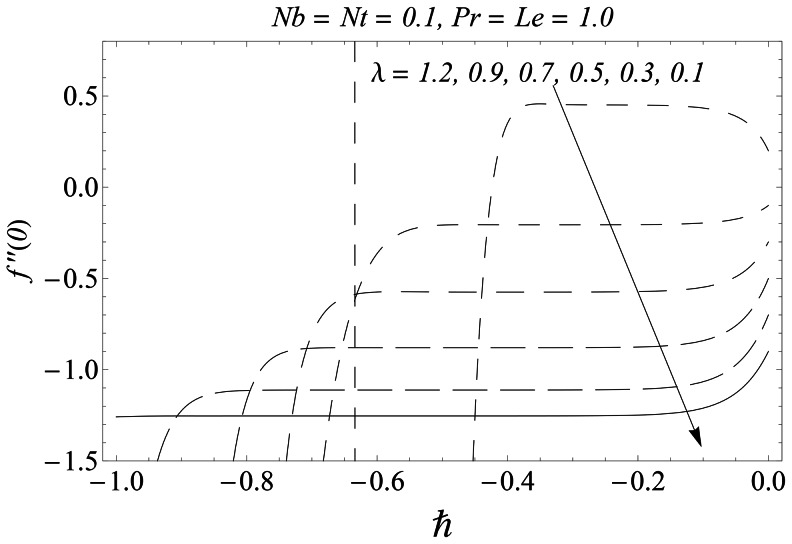
*h*∼curves for the function *f*.

**Figure 2:**
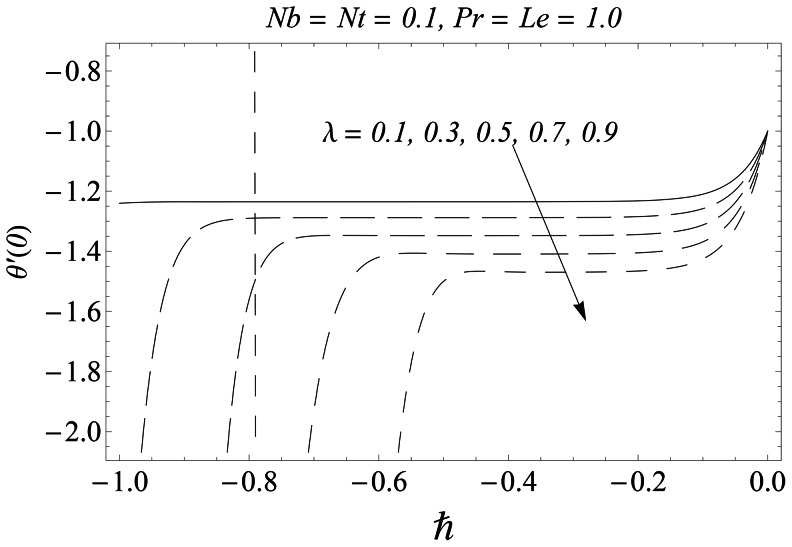
*h* ∼curves for the function *θ*.

**Figure 3:**
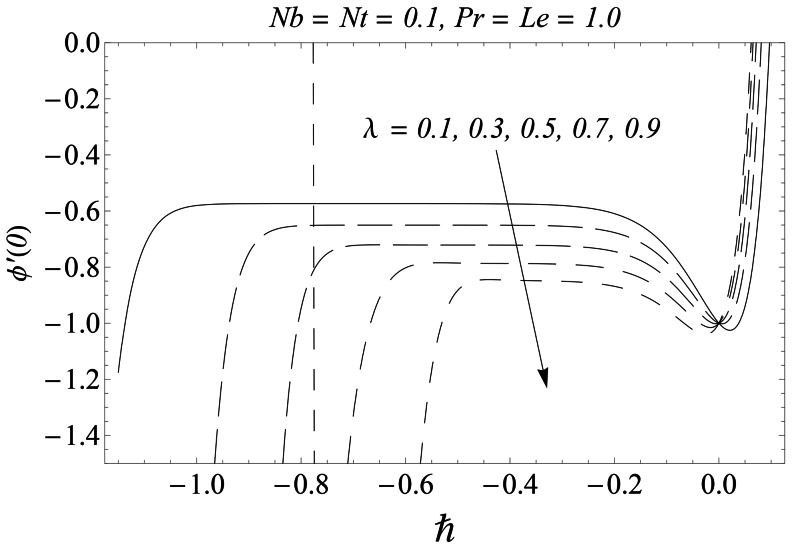
*h* ∼curves for the function 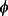
.

**Figure 4:**
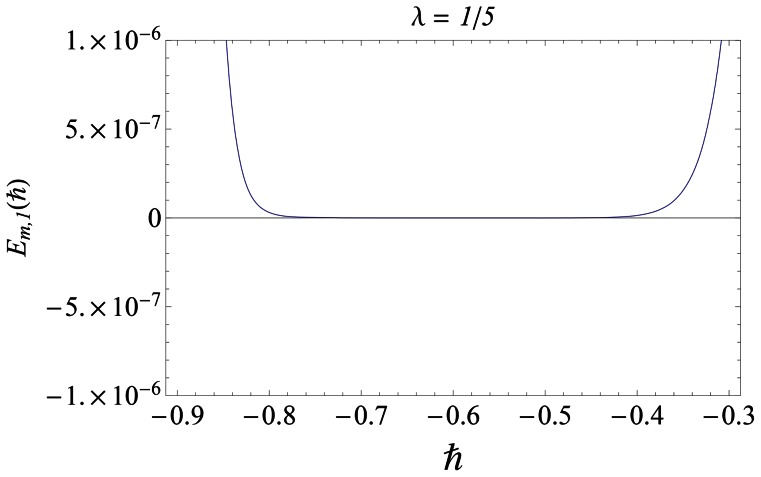
Averaged residual error for the function *f*.

**Figure 5:**
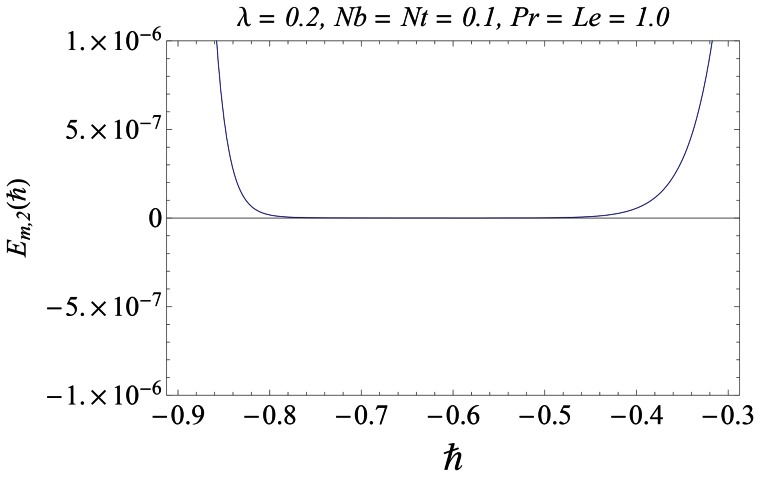
Averaged residual error for the function *θ*.

**Figure 6:**
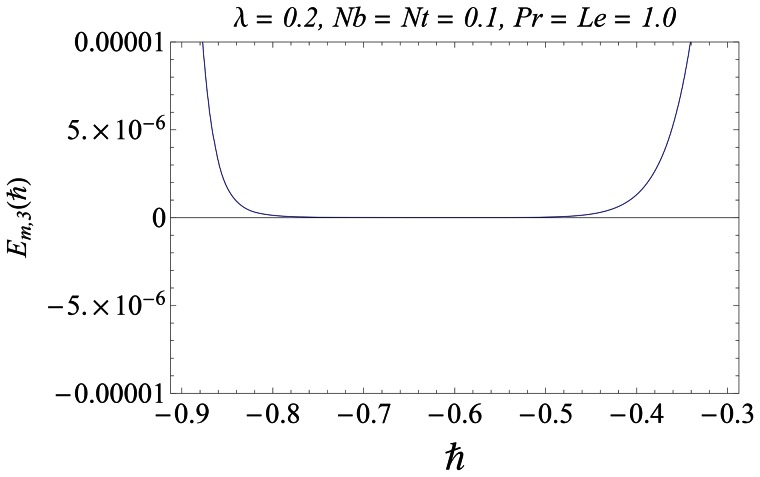
Averaged residual error for the function 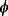
.

The auxiliary parameter 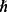 in Eqs. (26)–(28) has a key role in the convergence of HAM solutions (see Liao [30]). To select appropriate value of 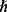 we have displayed the so-called 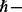
curves at 15th-order of approximations for different values of parameter 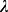 in Figs. 1, 2, and 3. Here the valid range of 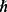 can be obtained from the flat portion of 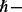
curves. The interval of convergence for 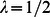 is 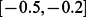. Further range of 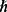 shrinks as we increase the values of 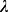. To see the accuracy of solutions we define the averaged residuals (see Ref. [36] for details) for the functions 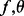 and 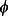 as
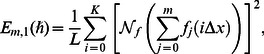
(37)

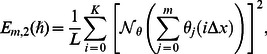
(38)

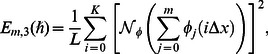
(39)where 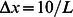 and 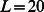. The averaged residual errors 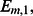 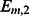 and 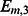 have been plotted versus 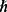 for some fixed values of parameters in Figs. 4, 5, and 6. From these figures we can obtain the best possible value of convergence-control parameter by calculating the minimum values of 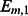, 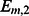 and 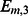.

### 3.2 Numerical method

Eqs. (8)–(10) subject to the boundary conditions 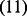 have been solved numerically by using the built in function bvp4c of the software MATLAB. This software uses the higher order finite difference code that implements a collocation formula (see Shampine et al. [37] for more details). It will be seen shortly that numerical solutions are in a very good agreement with the homotopy solutions for all the values of the embedding parameters.

## Numerical Results and Discussion

**Figure 7:**
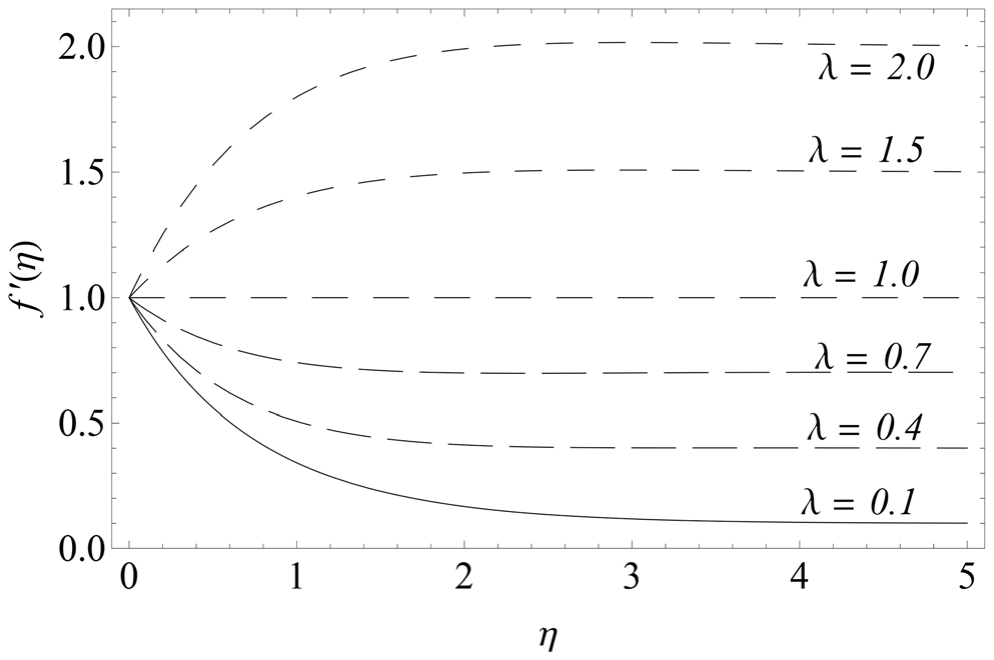
Influence of λ on 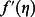.

**Figure 8:**
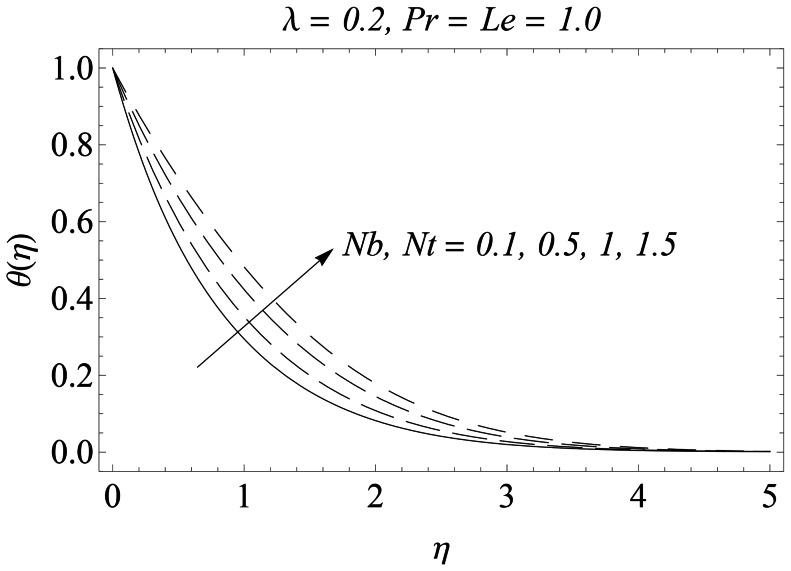
Influence of Nb and Nt on 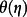.

**Figure 9:**
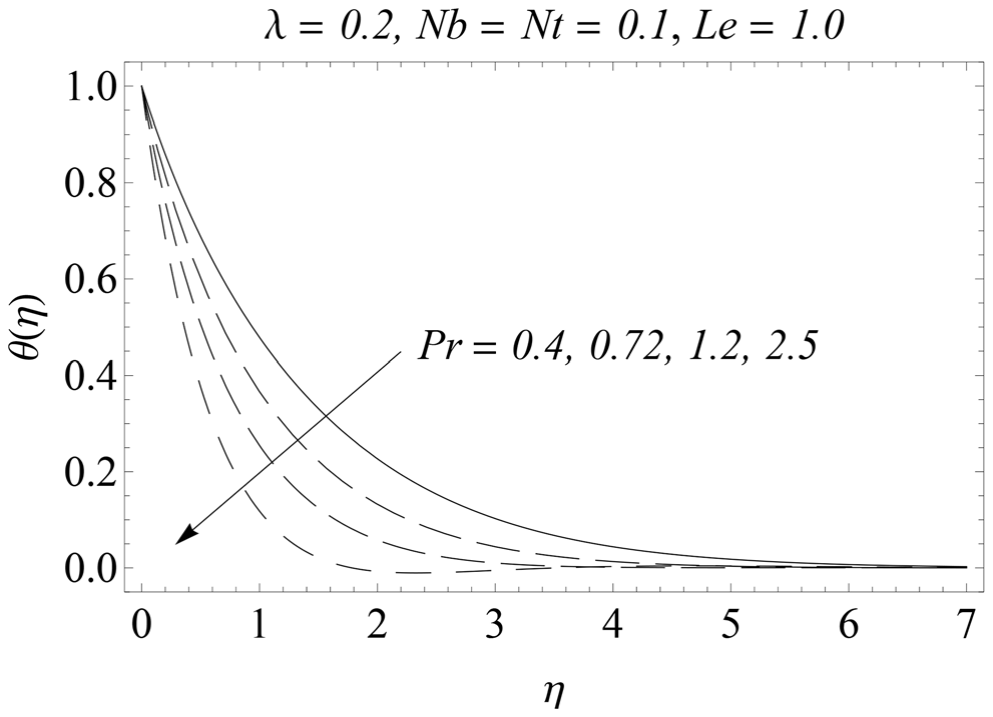
Influence of Pr on 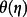.

**Figure 10:**
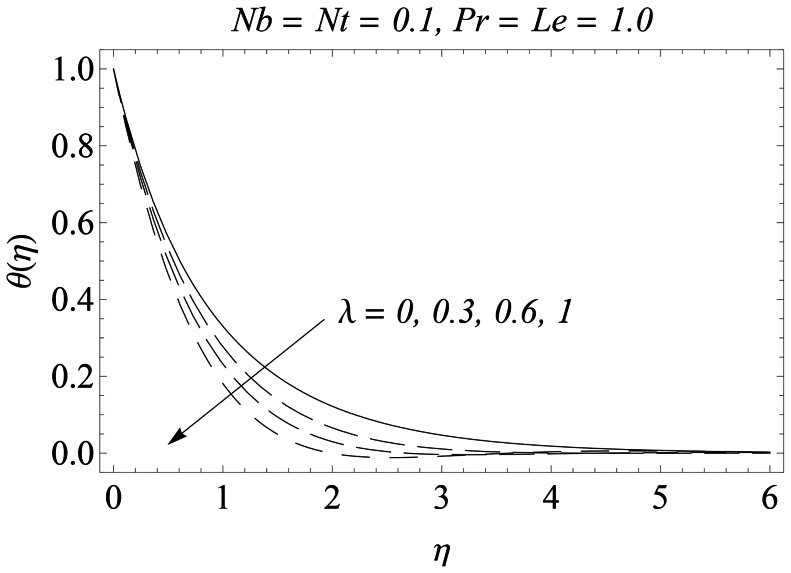
Influence of λ on 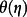.

**Figure 11:**
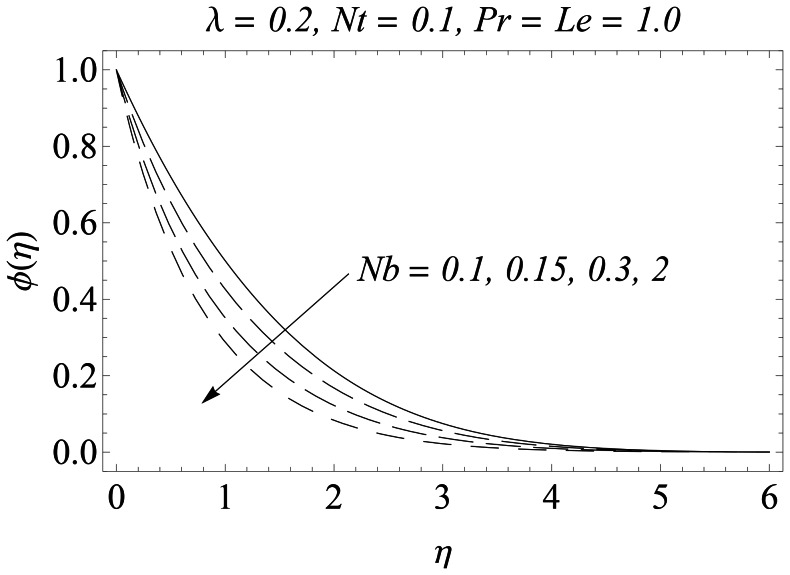
Influence of Nb on 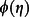.

**Figure 12:**
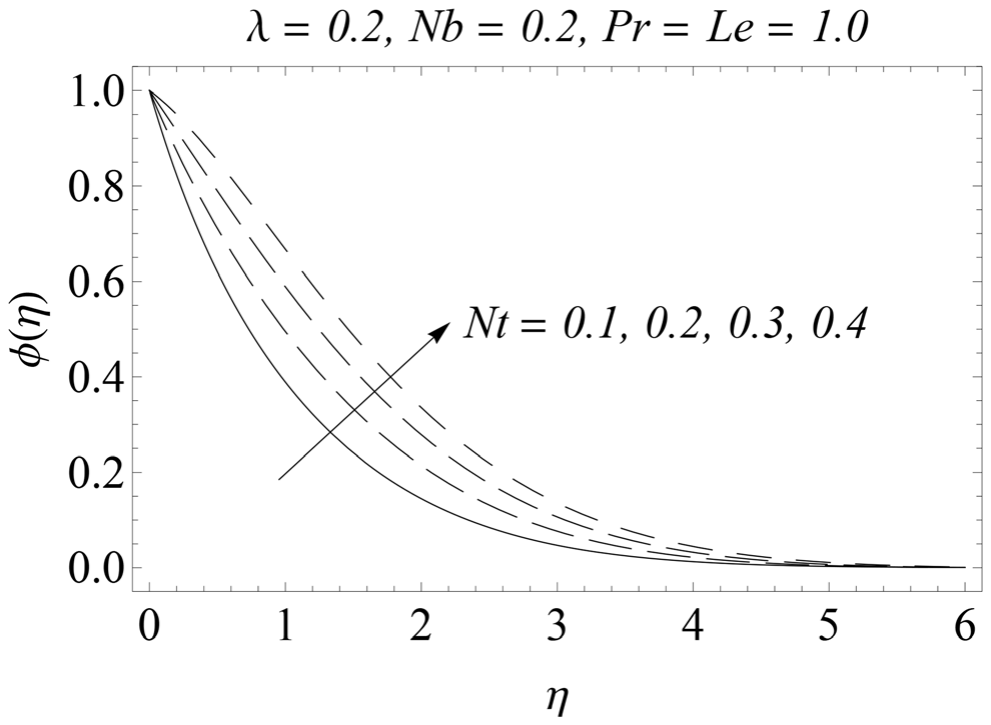
Influence of Nt on 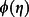.

**Figure 13:**
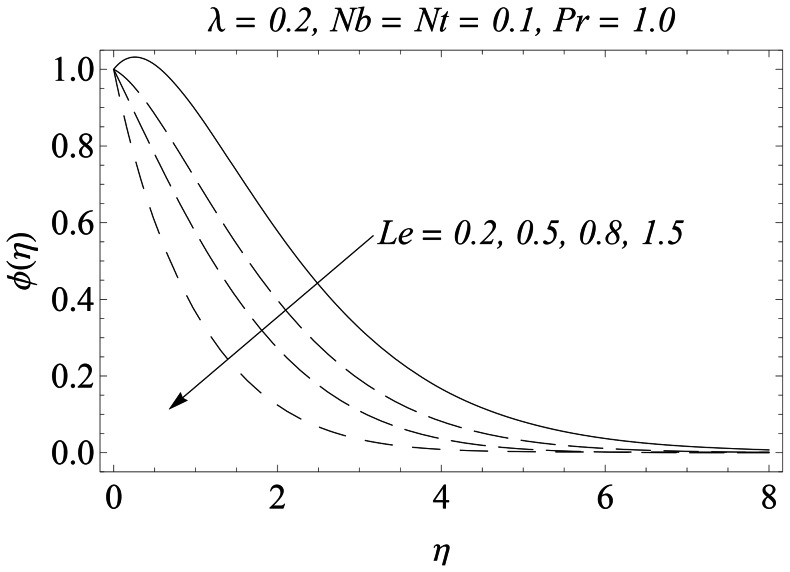
Influence of Le on 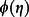.

**Figure 14:**
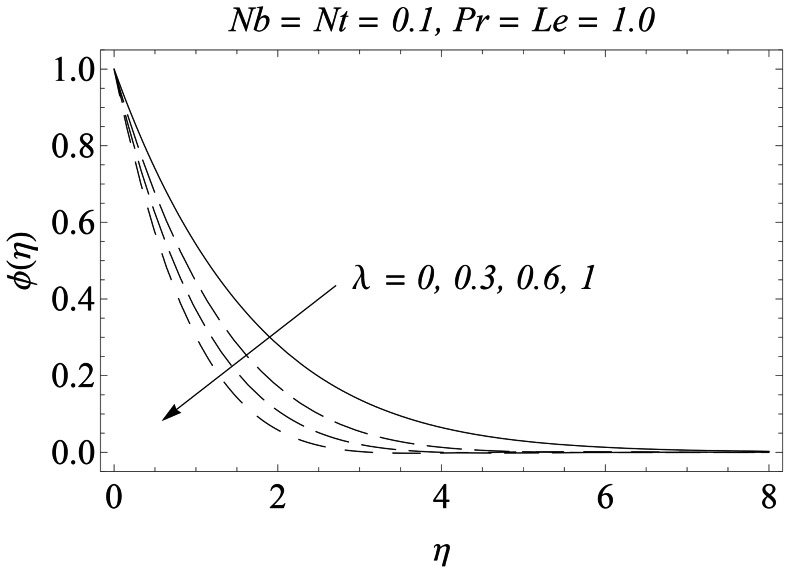
Influence of λ on 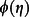.

The representative results for velocity, temperature and nanoparticles concentration are provided graphically and in tabular form. There is a considerable increase in the velocity with an increase in velocity ratio 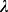 for some fixed values of parameters (see Fig. 7). It is evident from this figure that when 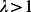, the thickness of the boundary layer decreases with the increase in 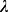. Here the straining motion near the stagnation region increases so the acceleration of the external stream increases which causes a reduction in the boundary layer thickness and as a consequence the horizontal velocity increases. On the other hand, when 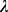 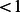, the flow has an inverted boundary layer structure. Here the sheet velocity 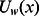 exceeds the velocity of external stream 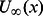. It is also noticed that boundary layer is not formed when 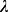 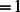. Fig. 8 is plotted to perceive the effects of Brownian motion and thermophoresis parameters on the temperature. There is a substantial increase in the temperature and the thermal boundary layer thickness with an increase in 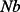 and 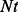. The growth in the thermal boundary layer thickness is compensated with smaller rate of heat transfer at the sheet. Fig. 9 portrays the behavior of Prandtl number 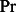 on the temperature 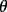. An increase in 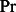 rapidly shifts the profiles towards the boundary causing a diminution in the thickness of thermal boundary layer. A bigger Prandtl number has a relatively lower thermal diffusivity. Thus an increase in 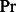 reduces conduction and thereby increases the variation in the thermal characteristics. As expected, the variation in the temperature is more pronounced for smaller values of 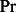 than its larger values. Fig. 10 depicts the effect of velocity ratio 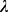 on the temperature 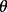. The temperature and the thermal boundary layer thickness decrease with an increase in 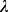. Fig. 11 plots the concentration function versus 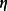 for different values of the Brownian motion parameter 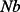. Here unlike the temperature 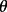, concentration boundary layer reduces as 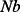 increases which thereby enhances the nanoparticles concentration at the sheet. Further we noticed that concentration 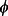 is only affected for the values of 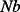 in the range 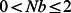. The influence of thermophoresis parameter 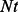 on the concentration boundary layer is noticed in Fig. 12. An abnormal increase in the concentration 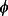 is found for a weaker Brownian motion (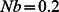). In fact an over shoot in the concentration function occurs as we gradually increase 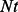. On the other hand, when the effect of Brownian motion is increased i.e 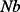 changes from 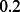 to 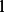, there is a little increase in the concentration 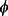 with an increase in 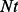. This outcome is attributed to the fact that an increase in 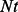 appreciably enhances the mass flux due to temperature gradient which in turn rises the nanoparticles concentration. The behavior of Lewis number 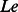 on the concentration field 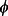 is presented in Fig. 13. As 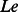 gradually increases, this corresponds to a weaker molecular diffusivity and thinner concentration boundary layer. In accordance with [27] the variation in 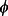 with 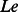 is prominent neat the stretching wall. Fig. 14 shows that the influence of 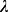 on the nanoparticles concentration 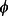 is virtually similar to that accounted for the temperature 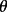.

**Figure 15:**
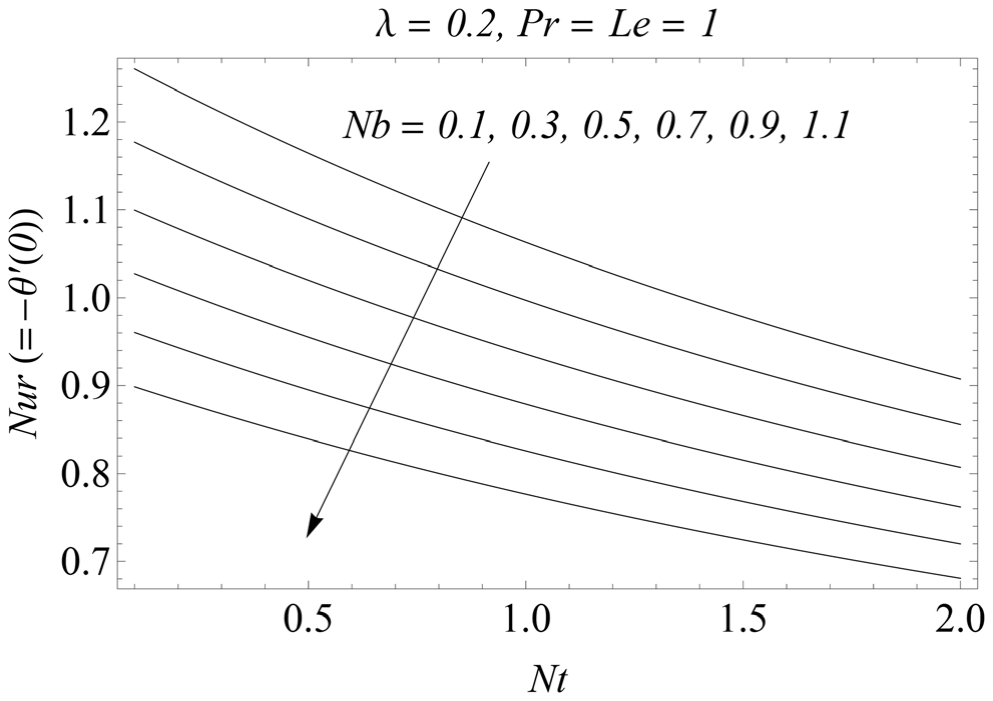
Influence of *Nb* and *Nt* on *Nur*.

**Figure 16:**
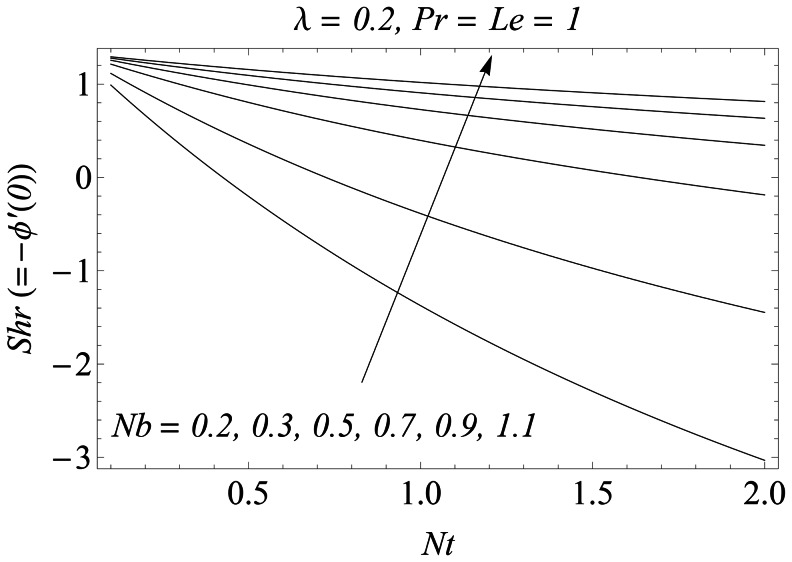
Influence of *Nb* and *Nt* on *Shr*.

**Figure 17:**
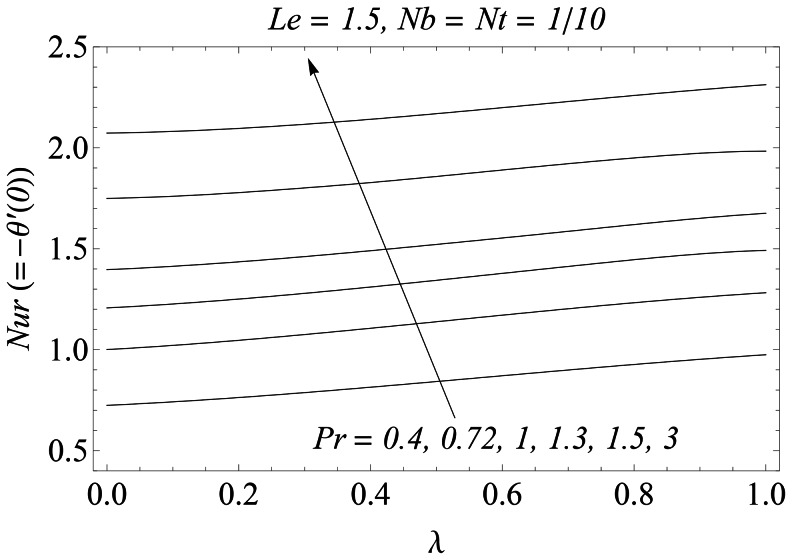
Influence of λ and *Pr* on *Nur*.

**Figure 18:**
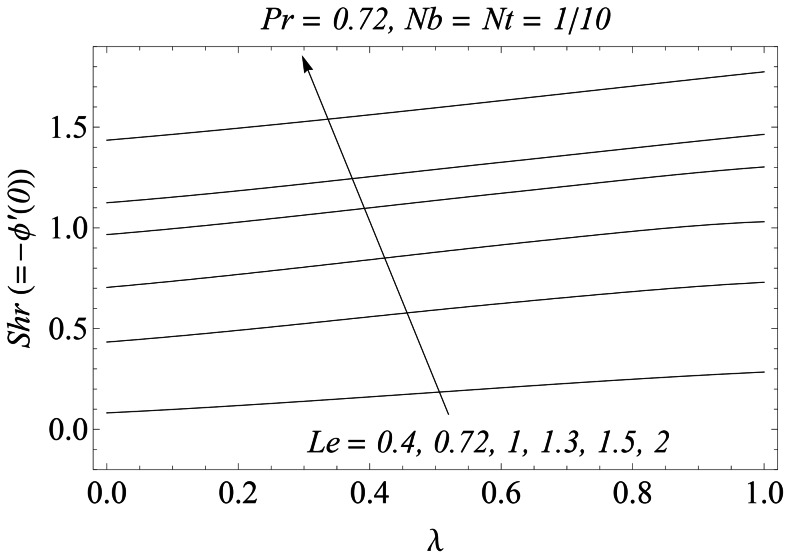
Influence of λ and Le on *Shr*.

Reduced Nusselt number 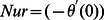 for different values of 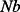 is plotted versus 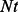 in the Fig. 15. It is observed that for a weaker thermophoretic effect, there is a significant decrease in the rate of heat transfer at the sheet with an increase in 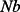. However when the strength of thermophoretic is increased i.e 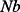 changes from 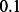 to 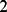 the absolute decrease in 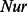 with an increase in 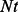 is negligible. This reduction actually occurs due to the excessive movement of nanoparticles from the stretching wall to the quiescent fluid. Fig. 16 shows the simultaneous effects of 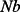 and 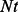 on the reduced sherwood number 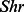. There is a slight increase in 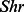 with an increase in 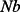 when the thermophoretic effect is weak. However this increase is significant when the thermophoretic effect intensifies. The variations of 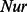 and 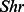 with the velocity ratio 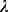 is sketched in the Figs. 17 and 18. The dimensionless heat and mass transfer rates at the sheet increase when 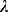 is increased. In Table 1 the dimensionless velocity gradient on the sheet is approximated for various values of 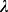. We observed that skin friction coefficient is reduced by assuming sufficiently large values of 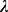. The numerical values of 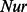 and 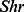 corresponding to different values of 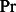 and 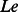 have been given in Table 2. It is clear from this table that numerical and analytical solutions are in a very good agreement. We noticed earlier that increase in 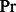 and 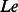 reduce the thermal boundary layer thickness and curves become steeper. The reduced Nusselt and Sherwood numbers, being proportional to the corresponding initial slopes, increase with an increase in 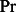 and 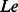 respectively.

**Table 1:** Numerical values of skin friction coefficient 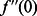 for different values of velocity ratio parameter 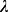.

λ	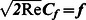 ′′(0)	
	HAM	Numerical
0	−1.281809	−1.281810
0∶1	−1.253580	−1.253580
0∶2	−1.195118	−1.195120
0∶5	−0.879833	−0.879835
0∶8	−0.397767	−0.397771
1∶2	0.451568	0.451571

**Table 2:** Numerical values of 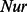 and 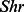 for different values of 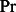 and 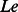 when 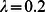, 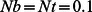 and 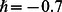.

Pr	*Le*	*Nur* = −θ′(0)		Shr = − 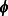 ′(0)	
		HAM	Numerical	HAM	Numerical
0.4	1	0.74994	0.74994	0.97397	0.97399
0.7		1.03426	1.03430	0.77875	0.77875
1.0		1.26024	1.26020	0.61269	0.61269
1.2		1.39072	1.39070	0.51328	0.51328
1.0	0.4	1.28094	1.28090	−0:11720	−0.11720
	0.7	1.26862	1.26860	0.29218	0.29218
	1.2	1.25588	1.25590	0.79694	0.76965
	1.5	1.25052	1.25050	1.04315	1.04320

## Conclusions

Flow of nanofluid in the region of stagnation-point towards an exponentially stretching sheet is studied. The developed mathematical problems have been solved for series solutions. A very good averaged residual error of about 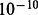 is achieved at only 15th-order of approximations in nearly all the cases. The numerical solutions are computed by the built-in solver bvp4c of the software MATLAB. Analytic and numerical solutions are found in excellent agreement for all the values of embedding parameters. It is observed that the velocity ratio 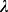 has a dual behavior on the momentum boundary layer. An increase in the strengths of Brownian motion and thermophoretic effects causes an appreciable increase in the temperature and the thermal boundary layer thickness. The current analysis for the case of regular fluid, which is not yet reported can be obtained by setting 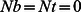.
